# Spatial Characterization of Hot Melt Extruded Dispersion Systems Using Thermal Atomic Force Microscopy Methods: The Effects of Processing Parameters on Phase Separation

**DOI:** 10.1007/s11095-013-1279-x

**Published:** 2014-02-20

**Authors:** Jonathan G. Moffat, Sheng Qi, Duncan Q. M. Craig

**Affiliations:** 1 School of Pharmacy, University of East Anglia, Norwich, UK NR4 7TJ; 2 Present Address: University College London School of Pharmacy, 29-39 Brunswick Square, London, UK WC1N 1AX

**Keywords:** hot melt extrusion, localized thermal analysis, solid dispersions, transition temperature microscopy

## Abstract

**Purpose:**

In this study we explore the use of nano-scale localized thermal analysis (LTA) and transition temperature microcopy (TTM) as a novel combined approach to studying phase separation in HME dispersions of cyclosporine A in Eudragit EPO.

**Methods:**

Modulated temperature differential scanning calorimetry (MTDSC), attenuated total reflectance FTIR spectroscopy, nano-LTA and TTM were performed on raw materials and dispersions prepared by hot melt extrusion (HME) and spin coating. For samples prepared by HME, two mixing temperatures (110°C and 150°C) and residence times (5 and 15 min) were investigated.

**Results:**

Spin coated samples showed an intermediate *T*_*g*_ for the mixed systems consistent with molecular dispersion formation. The HME samples prepared at 110°C showed evidence of inhomogeneity using MTDSC and FTIR, while those produced at 150°C h showed evidence for the formation of a single phase system using MTDSC. The nanothermal methods, however, indicated the presence of phase separated cyclosporine A at the higher preparation temperature while the TTM was able to map regions of differing penetration temperatures, indicating the presence of compositionally inhomogeneous regions in all but the high processing temperature/high residence time samples.

**Conclusions:**

TTM is a potentially important new method for studying phase separation and that such separation may remain undetected or poorly understood using conventional bulk analytical techniques.

## Introduction

The use of production methods involving hot melt extrusion (HME) has attracted considerable recent interest within the pharmaceutical industry (1). Attention has particularly focused on using HME to formulate drugs exhibiting poor water solubility characteristics (2–4), whereby incorporation of the drug into a water-soluble matrix can increase the dissolution rate and, potentially, the bioavailability. The commonly perceived ideal structure of such systems is a molecular dispersion, as theoretically such a structure ensures an homogenous drug distribution but also predicates that the drug is in a dissolved molecular state with no lattice energy to overcome prior to dissolution. However, such dispersions are well known to phase separate into either amorphous or crystalline drug regions and a number of studies have been conducted to facilitate prediction of such instability, including thermodynamic (solubility parameter, entropic and Flory-Huggins miscibility approaches (5, 6) and phenomenological approaches using thermal, spectroscopic and imaging methods (7). It has also been suggested that phase separation may manifest as conjugate mixes of drug and polymer rather than regions of pure material (8). More specifically, it has been suggested that phase separation may manifest as the formation of one or more binary mixes rather than pure materials, hence the notion that phase separation simply results in pure materials becoming spatially distinct may be open to challenge

In this study we investigate the use of novel thermal imaging approaches to identify phase separation, particularly in the context of correlating processing parameters to apparent miscibility. We have previously suggested (9) that the HME process may lead to elevated apparent solubility of the drug in the polymer compared to the equilibrium value due to the combination of temperature and mechanical force which in turn leads to the generation of a non-equilibrium molecular dispersion. It is therefore logical to suggest, and indeed it is widely recognized (10, 11), that the processing parameters used may have a profound effect on apparent miscibility. However detection of phase separation may be non-trivial, particularly at the early stages where the extent may be limited. For the characterization of solid dispersions, the most common approaches include (modulated) differential scanning calorimetry (12–15), Fourier-transform infrared (FTIR) (16, 17) and Raman (18, 19) spectroscopy and X-ray powder diffraction (XRPD) (20, 21). Furthermore, there are a number of techniques that may provide chemical information on the micro- to nano-scale, the most common of these being IR and Raman microscopy (22, 23).

Over the past two decades, techniques involving thermal atomic force microscopy (AFM) probes have been developed to provide spatially resolved thermal analysis (24–26). Thermal AFM probes involve supply of a voltage such that the temperature at the tip of the probe can be accurately controlled. These probes may also be employed in conventional AFM mode so as to provide topographical information, meaning that high resolution images can be generated and features on a surface can then be interrogated using thermal analysis. A typical measurement involves the application of a scanning voltage profile whilst the deflection of the probe is monitored; heat is transferred to the material beneath the probe causing it initially to expand which deflects the probe upwards. A thermal event such as melting or glass transition is subsequently observed as penetration of the probe into the surface, thereby allowing identification of components and/or physical forms via measurement of characteristic transition temperatures. This method is commonly known as localized thermal analysis (LTA).

A recent extension of these methods is known as transition temperature microscopy (TTM) whereby a series of these measurements is carried out over a grid pattern on a sample surface (27, 28), thereby allowing the generation of a two dimensional map of transition temperatures. This method therefore has the advantage of being able to determine the distribution of components across a complex surface in a systematic manner and with high datas capture as opposed to the selection of a limited number of user-selected points as described above. In this study the LTA and TTM approaches were employed to study dispersion systems containing a poorly water-soluble drug, cyclosporine A, in a water-miscible polymer, Eudragit EPO. This drug was chosen as part of a broader investigation to study the use of solid dispersion technology to enhance bioavailability of peptides (in this case a cyclic undecapeptide used as a post-operative immunosuppressant), while Eudragit EPO is a cationic polymethacrylate derivative widely used as an HME excipient due to its favorable thermomechanical properties and solubility at gastric pH. In particular, the effects of operating temperature and residence time within the extruder on structural microhomogeneity were investigated with a view to generating systems with varying levels of miscibility, thereby allowing evaluation of the characterization approaches. Here we study systems produced via both spin coating and HME so as to broaden the range of physical structures and surfaces produced using the same chemical components. We address the issues of whether thermal probe techniques may detect phase separation, how that phase separation may manifest and how the sensitivity of the thermal probe techniques compares to conventional thermal and spectroscopic methods.

## Materials and Methods

### Materials and Preparation Methods

Cyclosporine A (CsA) was purchased from Afine Chemicals Ltd. (China) and Eudragit EPO was kindly donated by Evonik (Germany). Thin film samples were prepared using a SCS G3P-8 lab-scale spin coater (Cookson Electronics, RI) with a spinning speed of 3000 RPM. Films of each component were prepared as well as film containing both components at a mass ratio of 1:1. For each film, a solution was made at a concentration of 10%w/v in ethanol. Droplets of the solution to a total volume of circa 500 μL were then placed onto a cover slide and allowed to spin for 2 min.

Hot melt extrusion (HME) samples were prepared on a Haake Minilab II extruder from Thermo Fisher (UK). The machine is a temperature controlled, twin screw, co-rotating system with a recycling loop which allows control over the residence time of the material in the system during processing. Samples were prepared at 110°C or 150°C as stated with a screw rotating speed of 50 rpm; preliminary studies using DSC and TGA indicated no evidence for degradation of the components using these conditions. No die was used at the orifice as this produced a sample with a relatively flat surface that would be more suitable for atomic force microscopy measurements. For all systems containing both drug and polymer, a drug loading of 50%w/w was used with an initial residence time of 5 min. Whilst this is recognized as a relatively high drug loading for HME processing, this value was selected as it allowed simpler investigation of both components using the selected characterization methods. The samples so produced were flat with a width of circa 5 mm and thickness of circa 1 mm. While the systems prepared at 110°C were cloudy, the 150°C samples were transparent.

### Physical Characterisation Approaches

All modulated temperature differential scanning calorimetry (MTDSC) measurements were carried out on the Discovery DSC from TA Instruments (Delaware, USA). Measurements were carried out over a temperature range 30 to 160°C with an overall heating rate of 2°C/min with a modulation of ±0.212°C every 40 s using standard Tzero pans (TA Instruments, Delaware, USA). Fourier-transform infrared (FTIR) measurements were carried out in attenuated total reflectance (ATR) mode. The spectrometer used was an IFS/66S system from Bruker Optics (UK) fitted with a Golden Gate ATR accessory from Specac Ltd. (UK). Measurements were carried out over a spectral range of 4,000 to 550 cm^−1^ with a total of 32 scans acquired at a resolution of 2 cm^−1^.

Topographic images were acquired with a Caliber atomic force microscope (AFM) from Bruker AFM (UK) equipped with an AN-200 Thermalever probe from Anasys instruments (USA). Localized thermal analysis was carried out using the same system as described above but equipped with a nanoTA2 controller also from Anasys Instruments. The tip of the probe is composed of a high resistance material and when a voltage is supplied by the nanoTA2 controller, the tip increases in temperature. A typical measurement is implemented by generating a topographical image of the sample surface using the AFM and then selecting a point of interest on the surface for interrogation. A voltage profile is then applied to the probe and the deflection of the probe monitored. As the probe heats the material directly beneath the probe, the material expands, forcing an upward deflection of the probe. As the temperature reaches a thermal transition such as a melting point, the material softens and the probe penetrates into the surface. At this point the temperature profile is stopped and the probe retracted. Prior to measurements the probe was calibrated for temperature using three polymeric standards with well-defined melting points. Once calibrated, measurements were carried over a range of 30 to 200°C at a heating rate of 10°C/s. An extension of this method is transition temperature microscopy (TTM) whereby a series of LTA measurements is carried out over a selected area. Once a transition temperature is detected the value is assigned to a color palette and subsequently a map is generated based on transition temperatures. TTM maps were generated on the VESTA system from Anasys instruments which is equipped with a microscope with a 10× objective which allows the selection of an area of interest for measurement. Over this area, LTA measurements with the same parameters as mentioned above were carried out over an area of 50 × 50 μm with a separation distance of 1 μm between measurements; the scale of scrutiny of individual measurements was in the region of 100 nm.

## Results

### Characterization of Pure Materials

**Fig. 1:**
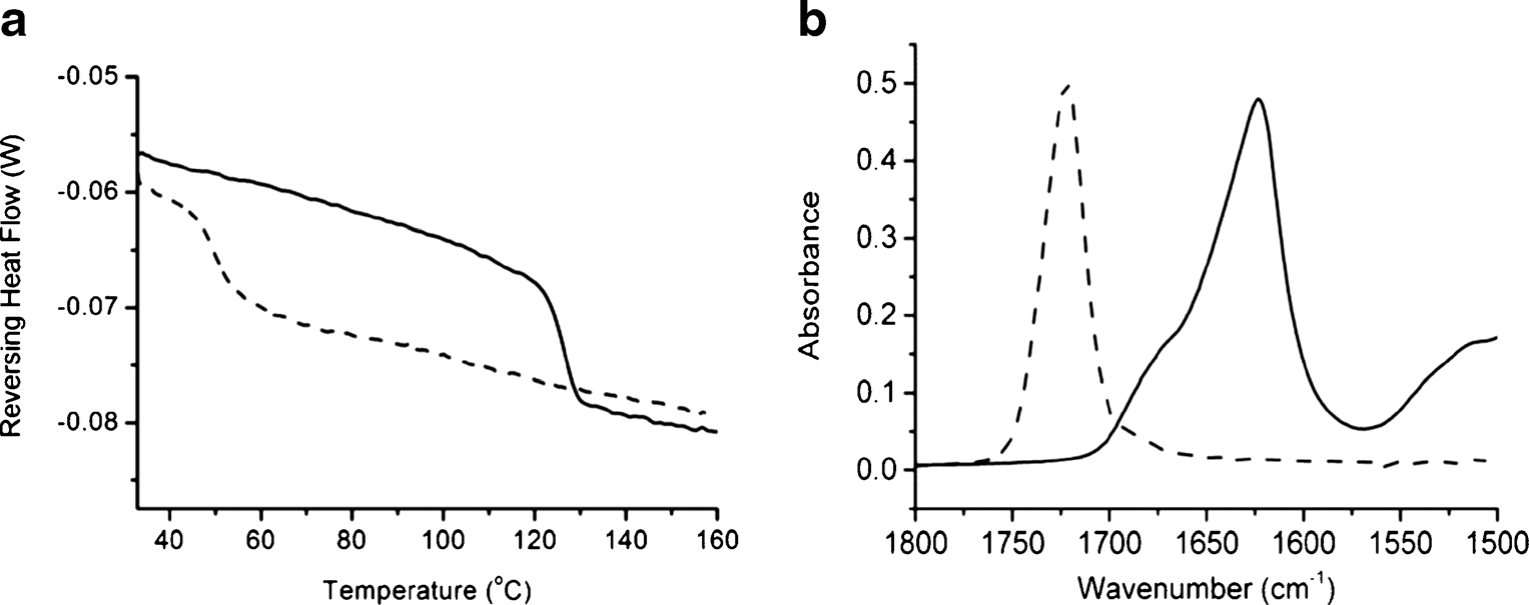
(**a**) Reversing heat flow MTDSC thermograms and (**b**) ATR-FTIR spectra of cyclosporine A (*solid line*) and Eudragit EPO (*dashed line*).

Prior to examination of samples prepared by HME, the pure materials were characterized by MTDSC, ATR-FTIR spectroscopy and LTA. Figure 1a shows the MTDSC reversing heat flow signals for CsA and Eudragit EPO as received from the supplier. Step changes in the reversing heat flow indicate the presence of a (putative) glass transition upon heating and the midpoints of the glass transition were observed at 126.1°C for CsA and 51.4°C for Eudragit EPO. These values are in good agreement with literature values (7, 9, 29) and indicate that both materials are in an amorphous form before processing by HME. However it should be emphasized that there is some uncertainty regarding the thermophysical behavior of CsA in that while some sources have attributed the heat capacity change to a glass transition (29), others have suggested that this material is liquid crystalline in nature (30). Irrespective of the explanation, the transition represents a means of identifying the presence of the peptide in phase separated form.

Understanding the thermal behavior of the raw materials was important for selecting operating conditions for HME processing; in particular a key consideration is that the material must be in a liquid-like state to allow movement inside the extruder barrel. Based on this requirement and the data in Fig. 1a, the operating temperatures investigated were 110°C and 150°C.

The ATR-FTIR spectra of CsA and Eudragit EPO are shown in Fig. 1b over the range 1,800 to 1,500 cm^−1^. Although spectra were recorded over the full mid-IR range this region is displayed as it focuses on an area where there is little spectral overlap. The band with peaks at ~1,625 cm^−1^ and ~1,670 cm^−1^ represent the carbonyl groups of CsA whilst the band with peak at ~1,725 cm^−1^ also represents the carbonyl groups of Eudragit EPO. For the mixed samples, investigation of the region of the spectrum allows simple visual inspection of spectra with regards to the relative amounts of each component within each system. For example, if multiple spectra are acquired on the same sample batch and the relative intensities of these bands varies then this can indicate the production of an heterogeneous batch.

To investigate the individual components using nano-LTA, it is desirable to measure the transition temperature of the powder form as received by the supplier. This proved to be difficult and resulted in unreliable data as samples are required to be stationary to allow contact with the probe without movement of the material. Other difficulties include the roughness of the particle, particularly given that the probe contact radius (~20 nm minimum, depending on penetration depth) will be relatively large in relation to the contours of the surface (31). An alternative approach is to prepare spin coated slides which involves dissolving the component in a particular solvent and then dropping an amount of this solution onto a spinning substrate. As the substrate spins, the solution spreads out and coats the surface allowing the solvent to evaporate rapidly, typically to produce an amorphous sample. On that basis, the spin coating approach was used as a simple method for providing data on the raw materials and, using mixed systems, as a comparator for the HME samples.

**Fig. 2:**
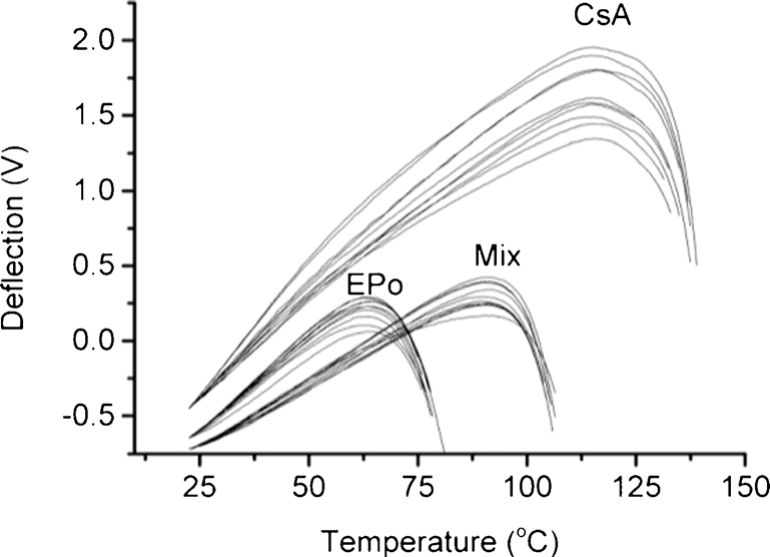
LTA profiles for spin coated slides consisting of Eudragit EPO (EPO), cyclosporine A (CsA) and a 50:50 mixture (Mix) of both components.

Figure 2 shows the LTA profiles for the spin coated individual components and the 50:50 mix. A consistent profile was observed for each sample and the observed transition temperature for each sample was 62.8 ± 0.8°C for EPO, 115.4 ± 0.8°C for CsA and 91.2 ± 1.2°C for the mixed system. For EPO alone the observed temperature was approximately 11°C higher than the midpoint of the glass transition as observed with MTDSC. This is a common observation when comparing LTA measurements with DSC responses (32); this is believed to be due to the transition observed with LTA being a softening response relative to the pressure exerted by the probe rather than necessarily the glass transition per se, combined with overshoot resulting from the rapid heating rates used for LTA experiments. It is interesting to note that the observed transition for the lower molecular weight CsA compares well with the onset of its glass transition observed with MTDSC. The transition noted for the mixed system is indicative of molecular miscibility, with the intermediate value noted compared to the raw materials being compatible with the well-known Gordon-Taylor approach which predicts such behavior for plasticized materials (33). The *T*_*g*_ of a miscible system (*T*_*gmix*_) is predicted as:

1 $$ {T}_{gmix}=\frac{w_1{T}_{g1}+K{w}_2{T}_{g2}}{w_1+K{w}_2} $$

where *w*_*1*_ and *w*_*2*_ are the weight fractions of components 1 and 2 respectively, *T*_*g1*_ and *T*_*g2*_ are the respective measured glass transition temperatures and *K* is the ratio of the free volumes of the 2 components which can be simplified to (34)

2 $$ K\approx \frac{\rho_1{T}_{g1}}{\rho_2{T}_{g2}} $$

where *ρ*_*1*_ and *ρ*_*2*_ are the true densities of each component. Using the measured *T*_*g*_ values mentioned earlier, the density of EPO (35) as 1.11 g/cm^3^ and an approximated value of 1.20 ± 0.1 g/cm^3^ for the density of CsA then the predicted *T*_*g*_ of a miscible system will be 83.8 ± 1.6°C which compares well with the transition observed with LTA (Fig. 2).

### Effects of Processing Temperature and Residence Time on HME Disperse Systems – Bulk Thermal and Spectroscopic Studies

To investigate the effect of processing temperature on the production of HME solid dispersions, two temperatures were chosen (110°C and 150°C) on the basis of their being above the transition temperature of the Eudragit, thereby ensuring liquid-like properties. Operation at lower temperatures would have the benefit of limiting the possibility of thermal degradation and also reduced operating costs, hence there is great interest in minimizing the temperature used for the process. It should also be noted that the sample produced at 110°C produced an opaque sample indicating that the drug had not dissolved fully into the polymer or had not remained dissolved during the extrusion process. It is therefore suggested that the two temperatures represent scenarios whereby the drug is likely to be in different forms within the dispersion, thus providing a suitable system for assessing the characterisation methods.

**Fig. 3:**
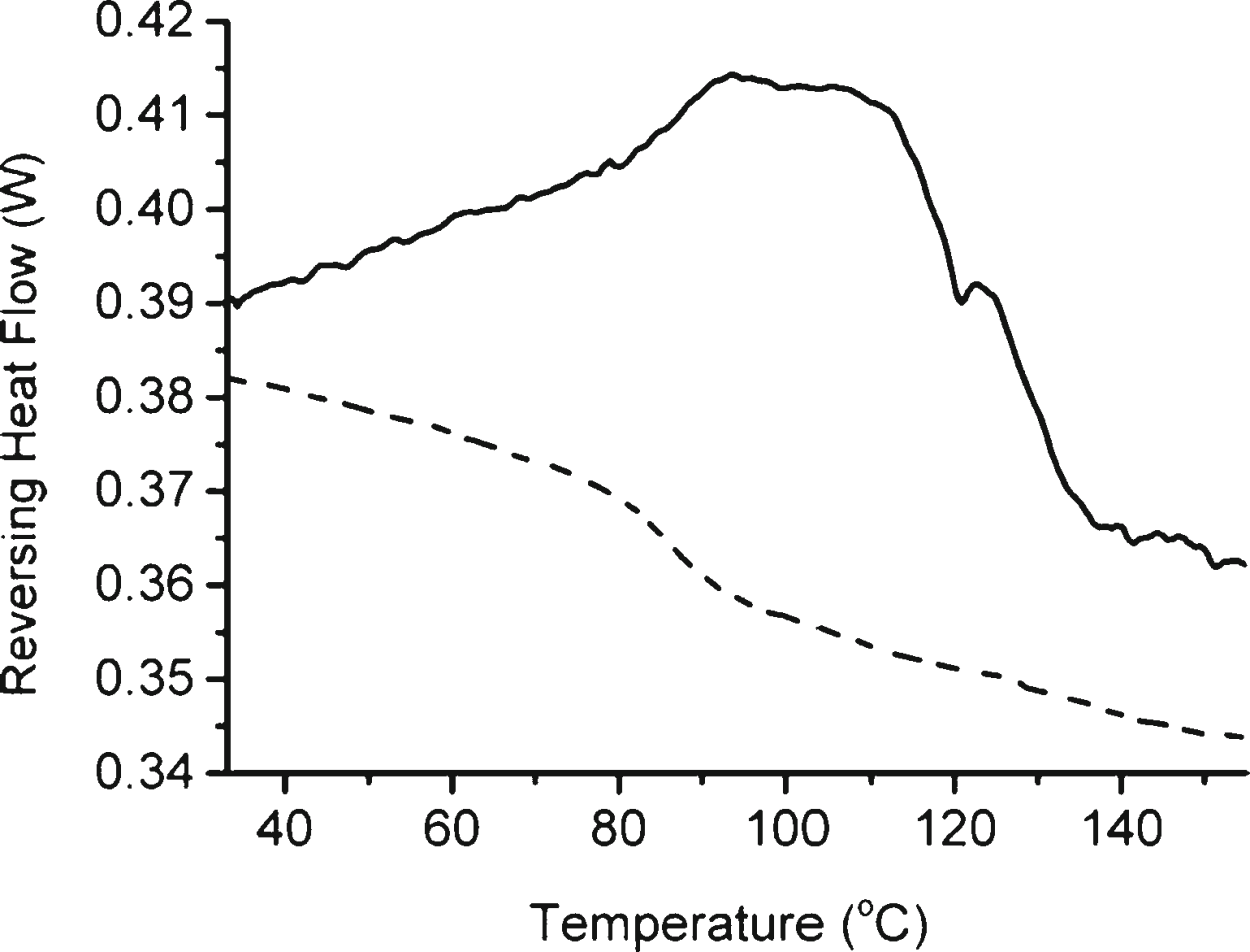
MTDSC reversing heat flow profile of 50:50% w/w CsA in EPO HME dispersions prepared at 110°C (*solid line*) and 150°C (*dashed line*).

Figure 3 shows representative MTDSC responses of systems prepared at both temperatures. For MTDSC, the observation of a single glass transition would indicated a single phase system and hence the creation of a solid dispersion. For the system generated at 110°C the thermal response in Fig. 3 is difficult to interpret as, while there appears to be a glass-like transition at circa 120°C, there was a high degree of baseline noise. Repeat measurements of this sample showed similar behavior (glass-like transition and noise) but the reproducibility of the response was poor. It is interesting to note that no clear transition was seen corresponding to the EPO, indicating that the system is unlikely to consist of a simple mix of the two separated components. The thermal response of the system formed at 150°C shows a clear single glass transition. Multiple repeats of this sample with MTDSC showed an average *T*_*g*_ of 85.7 ± 1.5°C which agrees well with the Gordon-Taylor prediction in the previous section, indicating a miscible system with a homogenous distribution.

**Fig. 4:**
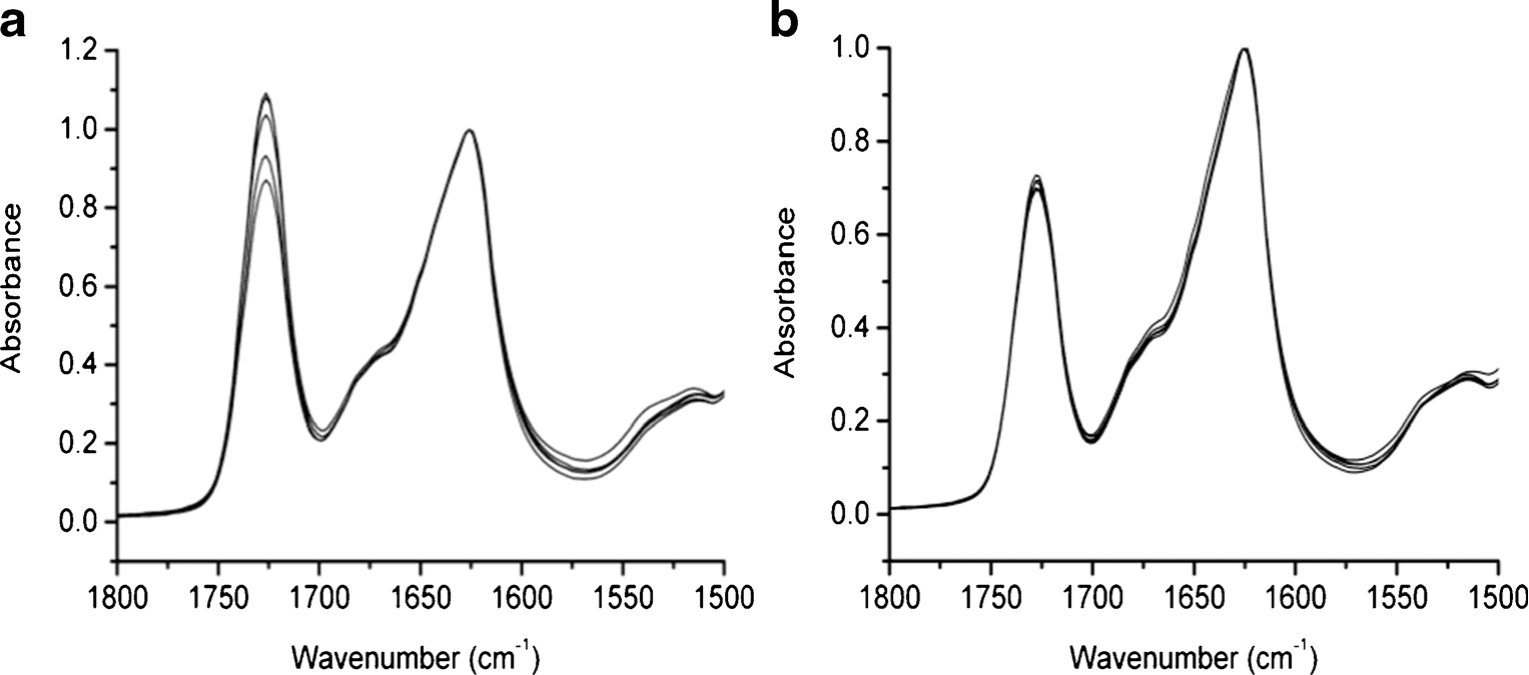
ATR-FTIR spectra of HME samples composed of EPO and CsA at a ratio of 50%w/w prepared at (**a**) 110°C and (**b**) 150°C. Five sample spectra are shown on each plot from the same batch.

Multiple ATR-FTIR spectra of each system produced at 110°C and 150°C are shown in Fig. 4 and are normalized to the band at ~1,625 cm^−1^ for comparison. As mentioned in the previous section, the bands centered at 1,725 and 1,625 cm^−1^ are due to contributions from Eudragit and CsA, respectively, with no evidence of peak shifts (and hence molecular interactions) observed. Comparison of the uniformity of the relative intensities of the 2 bands will give an indication of sample heterogeneity with respect to component distribution. Figure 4a shows that there is a significant variation in the relative amounts of each component for the system produced at 110°C whilst the systems produced at 150°C showed little variation. It is interesting to note that the ratio of the EPO band to CsA is higher for the system produced at 110°C. As the ATR technique probes the outer 1-2 μm layer of a sample, the spectra indicate that there is a higher concentration of polymer at the surface for the system produced at the lower temperature. This is likely to be due to the polymer forming an outer film on the extrudate due to its greater mobility; similar effects have been noted for felodipine dispersions in Eudragit EPO (7).

### Effects of Processing Temperature and Residence Time on HME Disperse Systems – Thermal Probe Investigations

The results thus far indicate that the systems prepared at 150°C produced an homogenous molecular solid dispersion, while the 110°C systems showed evidence of both phase separation and non-homogeneity within the extrudate samples. On this basis thermal probe methods were employed to gain information on a spatially resolved basis.

**Fig. 5:**
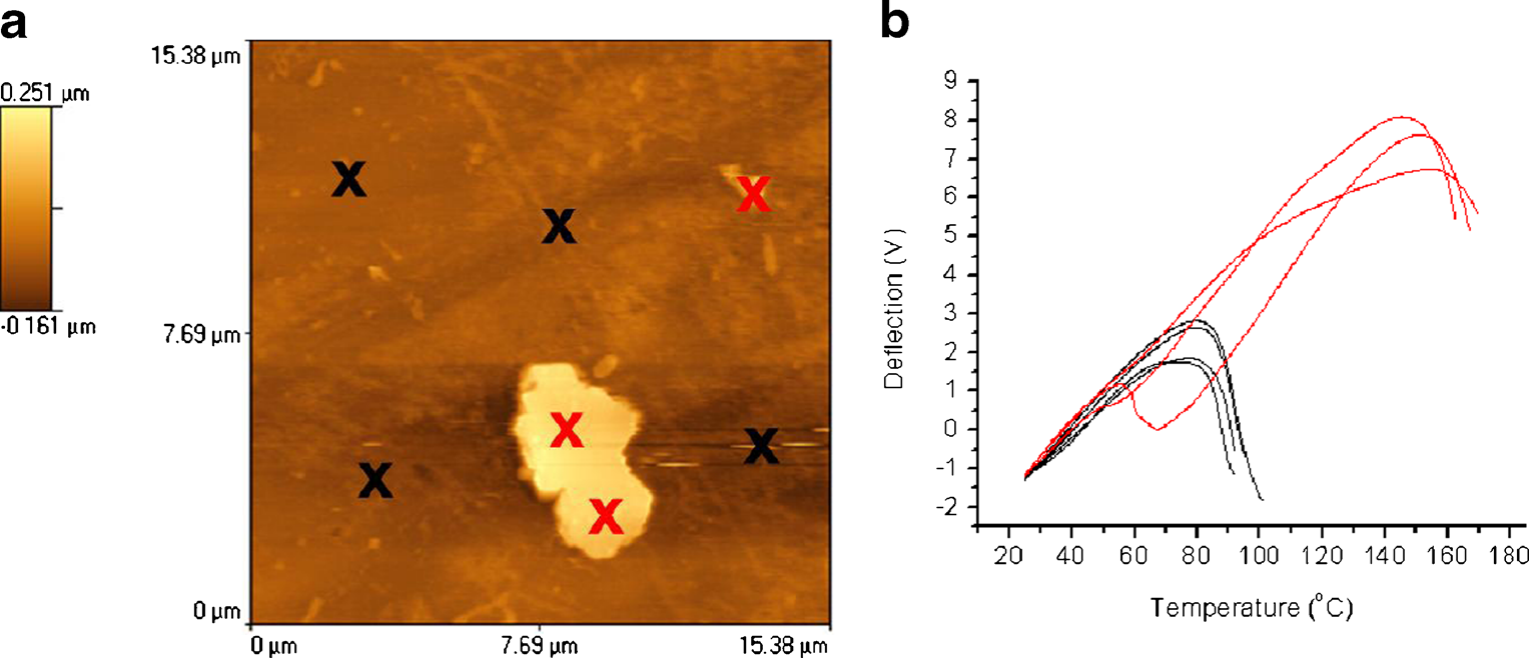
(**a**) AFM topographic image of a 50% w/w CsA in EPO HME sample produced at 150°C and (**b**) corresponding LTA measurements acquired from locations marked with an X on (**a**).

Initially AFM topographic images were acquired to identify any surface features of interest. For the system formed at 110°C, it was not possible to acquire satisfactory images as the height of surface features was higher than the z-range of the AFM scanner. For the system processed at 150°C, a smooth surface was produced that could be imaged with AFM. The majority of images acquired on this sample were relatively featureless, but occasionally some particles with a diameter of 3–5 μm were observed. Figure 5a shows a representative image of one of these particles on the surface of the extrudate. To determine the nature of this particle, single point LTA measurements were carried out at locations marked on the surface. Figure 5b shows the corresponding LTA responses. Whilst the majority of profiles show a thermal event at ~90°C which agrees well with the *T*_*g*_ observed in Fig. 3, there are a lower number with transitions at ~120°C which correspond well with the *T*_*g*_ of CsA. This indicates that even though MTDSC indicated miscibility, there are some regions of phase separation in the 150°C samples.

**Fig. 6:**
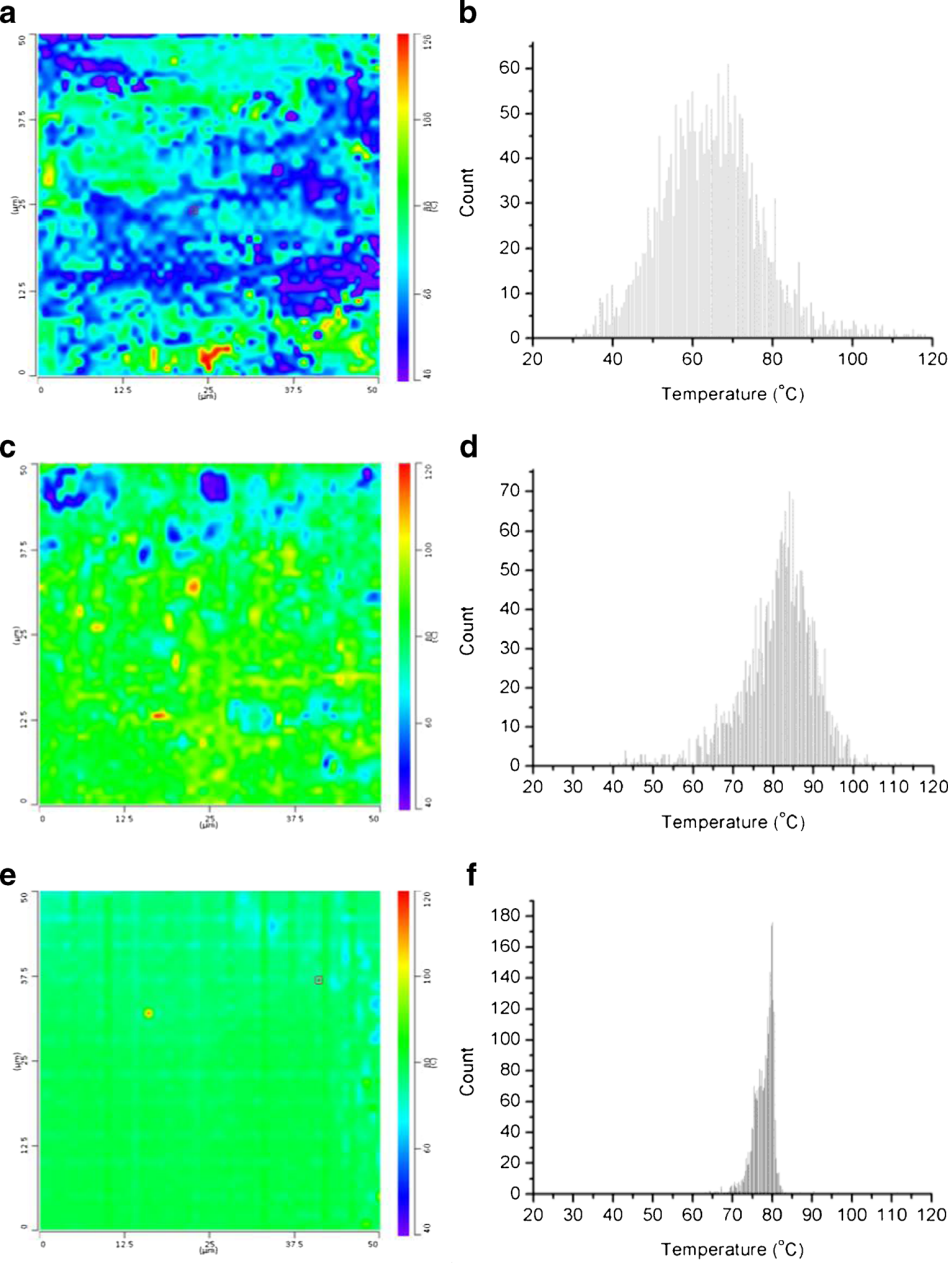
Transition temperature microscopy maps and corresponding histograms of 50% w/w CsA in EPO HME systems produced after a residence time of 5 min and prepared at 110°C (**a** and **b**, respectively) and 150°C (**c** and **d**, respectively). TTM map and corresponding histogram of a HME system prepared at 150°C with a residence time of 15 min (**e** and **f**, respectively).

The distribution of components in each system was further investigated with TTM as this provides a systematic approach to the user-selected measurements shown in Fig. 5. With TTM it is also possible to measure rough samples as the measurements are not based on the scanning movement of the probe and instead involve lateral movement when the probe is not in contact with the surface, hence the 110°C samples could be analysed successfully. Figure 6a and c show the TTM maps for a sample produced at 110°C and 150°C respectively whilst Fig. 6b and d show the corresponding histograms of the measured transitions.

For the system prepared at 110°C it is clear that sample surface is heterogeneous with a wide distribution of transition temperatures. It is interesting to note that the mode of the distribution is in the region of 67°C, and while there are points which correspond reasonably well with both the drug and polymer individually the majority of measurements are intermediate between the two. This heterogeneity, and the lack of clustering around the two individual material *T*_*g*_ values, suggests a more complex pattern of phase separation than simple spatial separation of the pure materials and may reflect phase separation via compositional variation of binary material, as suggested by Meng *et al*. (8). Alternatively, the scale of scrutiny of the instrument (circa 100 nm) may be such that the probe is landing on interfaces between phase separated regions which may lead to intermediate transition temperatures being recorded.

By increasing the operating temperature to above the *T*_*g*_ of the drug (150°C) a significantly more homogenous system was created. Whilst the majority of measurements are centered around the *T*_*g*_ of the molecular dispersion, the distribution is relatively broad and there are still areas of the order of ~10 μm^2^ showing non-dispersed drug and polymer, indicating that the distribution is not homogenous at a micron to sub-micron level.

As the data shown in Fig. 6a to d did not indicate a homogenous system, the residence time of the components in the extruder was increased to allow complete mixing. Figure 6e and f show the TTM map and corresponding histogram of a system produced at 150°C after 15 min residence time. The TTM map shows a significant improvement in the homogeneity with a narrow distribution of transition temperatures (Fig. 6e and f).

## Discussion

The results shown in Fig. 6 demonstrate the capabilities of thermal AFM probes, and TTM in particular, to determine the homogeneity within a system produced by HME and hence assist the establishment of optimal parameters for the production of a solid dispersions. In the case here, conventional techniques such as FTIR spectroscopy and MTDSC indicated the production of a molecular dispersion using a residence time of 5 min at a temperature of 150°C. By using TTM, it was shown that these process parameters did not show an evenly dispersed system at the μm scale and that an increased residence time was required for more complete homogeneity. Consequently, we would argue that TTM represents a potentially important method of determining whether a system is truly homogeneous following preparation of the HME dispersion.

Transition temperature microscopy was found to be particularly useful in investigating the system formed at 110°C, where clear evidence of inhomogeneity was generated, reinforced by the physical appearance and the MTDSC and spectroscopic data. Despite the benefits of these localized thermal techniques, there are some drawbacks that should be highlighted. With any technique that is used to identify components there is the need for reference materials as standards. In this study, spin-coated films were introduced as a reference for localized thermal techniques. As has been described previously (31), localized thermal methods can be influenced by surface topography. Spin-coated films produce a surface with a roughness in the order of a few nanometers and hence minimize topographical effects on measurements (36). Furthermore, with any imaging technique used to map components there is the issue of acquisition time. Due to the delicacy of these probes and the nature of the measurement, a relatively slow approach to the sample surface is required resulting in a total acquisition time per measurement of ~30 s meaning that images here were generated over a time period of approximately 15 h, considerably longer than spectroscopic imaging techniques. This acquisition time could be reduced by reducing the retraction height after measurement but would only be advisable if the sample was relatively flat.

## Conclusion

In this study, methods involving thermal AFM probes were employed to characterize and map the distribution of components within a solid dispersion prepared by hot melt extrusion. These methods were also compared to standard bulk techniques MTDSC and ATR-FTIR spectroscopy. Initially the effect of processing temperature was investigated and it was confirmed by both MTDSC and TTM that a molecularly dispersed system was not produced at a temperature below the *T*_*g*_ of the drug. MTDSC indicted the presence of a single phase system at a processing temperature above the *T*_*g*_ of the drug whilst TTM indicated a relatively poorly mixed system at a micrometer level. By increasing the residence time of the components in the extruder a significantly more homogenous surface was observed which would imply the production a molecular dispersion. These findings not only demonstrate the importance of using spatially resolved techniques for solid dispersion characterisation but also highlight the limitation in using standard bulk techniques.

## Abbreviations

AFM: Atomic force microscopy
ATR: Attenuated total reflectance
CsA: Cyclosporine A
FTIR: Fourier transform infrared
HME: Hot melt extrusion
LTA: Localised thermal analysis
MTDSC: Modulated temperature differential scanning calorimetry
TTM: Transition temperature microscopy
XRPD: X-ray powder diffraction
*Tg*: Glass transition temperature
*w*: Weight fraction of component
*ρ*: Density of component
